# Post-COVID-19 syndrome symptoms after mild and moderate SARS-CoV-2 infection

**DOI:** 10.3389/fmed.2022.1017257

**Published:** 2022-10-03

**Authors:** Lou’i Al-Husinat, Mokeem Nusir, Haitham Al-Gharaibeh, Amer A. Alomari, Mahmoud M. Smadi, Denise Battaglini, Paolo Pelosi

**Affiliations:** ^1^Department of Clinical Medical Sciences, Faculty of Medicine, Yarmouk University, Irbid, Jordan; ^2^Department of Neurosurgery, San Filippo Neri Hospital/ASLRoma1, Rome, Italy; ^3^Department of Mathematics and Statistics, Jordan University of Science and Technology, Irbid, Jordan; ^4^Anesthesia and Intensive Care, San Martino Policlinico Hospital, IRCCS for Oncology and Neuroscience, Genoa, Italy; ^5^Department of Surgical Sciences and Integrated Diagnostics (DISC), University of Genoa, Genoa, Italy

**Keywords:** post-COVID-19 syndrome, SARS-CoV-2 infection, chronic COVID-19 syndrome, mood disturbance, post-acute sequelae of SARS-CoV-2 infection

## Abstract

**Background:**

Post-COVID-19 Syndrome (PCS) is characterized by residual symptoms following the initial recovery from severe acute respiratory syndrome coronavirus-2 (SARS-CoV-2) infection. The prevalence of PCS is known to be the highest among severe and critical forms of the disease. However, the occurrence and risk factors for PCS after mild or moderate SARS-CoV-2 infection has not been extensively investigated.

**Methods:**

Online and offline *via* both paper or mailed questionnaires distributed among Jordan collected between 1st and 21st August 2021, including a total number of 800 respondents, of whom 495 had previous mild to moderate COVID-19 infection. The Newcastle post-COVID syndrome Follow-up Screening Questionnaire was modified, translated, and used as a standard instrument for data collection regarding psychological, medical, and socio-economic symptoms post-infection. The primary outcome was the prevalence of PCS after mild to moderate COVID-19 in Jordan. Secondary outcome was the identification of PCS risk factors.

**Results:**

The most common PCS symptom was mood disturbance followed by fatigue, anxiety, and myalgia. Female gender significantly increased the risk for multiple PCS symptoms. Age < 30 years was found to be an independent risk factor for myalgia (*p* = 0.001).

**Conclusion:**

PCS is highly prevalent among COVID-19 survivors in Jordan, especially in females and patients with comorbidities. Planning physical and mental rehabilitation services is recommended for those patients with PCS symptoms after mild to moderate COVID-19 infection.

## Introduction

Infection from severe acute respiratory syndrome coronavirus (COVID-19) may present different clinical presentations and degrees of severity. Clinical presentation may vary from an asymptomatic disease to an infection of the upper respiratory tract or a severe disease with potential for multiple organ involvement, high morbidity, and mortality (1). COVID-19 survivors may experience long-lasting psychological, medical, and socio-economic sequelae. Several definitions of post-COVID-19 sequelae have been proposed, including post-COVID-19 syndrome (PCS), long COVID-19, chronic COVID-19, and long haulers (2). Nevertheless, the exact definition, mechanism, and clinical impact of these symptoms are still unclear. Patients with the most severe form of the disease and requiring hospital and/or Intensive Care Unit (ICU) admission are at higher risk for developing PCS and long-term symptoms. Individuals hospitalized because of COVID-19 present high levels of disability, dyspnea, dysphagia, and dependence for both activities of daily living (ADL) and instrumental activities of daily living (IADL) (3). New illness-related fatigue was the most common reported symptom, followed by breathlessness, smelling and taste dysfunction and psychological distress (4–6). The occurrence of PCS and related symptoms after the mild to moderate COVID-19 infection remains to be elucidated. We therefore performed an online survey in Jordan with the aim to investigate the prevalence of PCS in non-hospitalized subjects with mild to moderate COVID-19 infection not requiring respiratory support. We also assessed the impact of age and gender on PCS as well as potential individual risk factors.

## Materials and methods

This study was performed between 1st and 21st August 2021, in Jordan. The study protocol was approved by the International Review Board (IRB) of Yarmouk University number IRB/2022/9. Consent for participating was given by responding to the questionnaire. In the cover letter of the online-based survey, the participants were informed about the purpose of the study, ensured confidentiality, and the voluntary nature of the study; the possibility of withdrawing from the study at any time was emphasized. The questionnaire aimed to determine the pattern of PCS symptoms among mild and moderate COVID-19 survivors in Jordan and to identify subjects who may benefit from a medical and psychological multi-disciplinary assessment.

### Study participants and selection criteria

A cross-sectional self-administered-online and offline-based questionnaire study involved 800 participants from all governorates of Jordan and different educational as well as governmental institutions. Inclusion criteria were previous SARS-CoV-2 infection confirmed with a positive polymerase chain reaction PCR result and currently being at least 10–12 weeks from the onset of acute illness. Patients who have been admitted to a hospital or required respiratory support of any kind were excluded from the analysis, therefore, we only analyzed outpatient survivors with history of mild to moderate illness.

### Assessment procedure and material

A standard questionnaire composed of 23 questions was modified and translated into Arabic and converted into a web-based survey using Google Forms Application. A modified version of the Newcastle post-COVID syndrome Follow-up Screening Questionnaire was used as a standard instrument, it was applied as one of the long-COVID SNOMED-CT codes which were developed and released in the UK in November 2020 to support clinical care and implementation of NICE guidance (7), to be carried out 10–12 weeks after the acute illness (Supplementary material).

The questionnaire distribution was divided into three different parts. The first part related to demographic information, the second part was about the clinical data and the third focused on other symptoms clinically relevant for the patient.

General and neurological symptoms included myalgia, fatigue, change/loss of smell and taste, weakness, and weight loss in 3 months. Psychological symptoms included sleep disturbances, nightmares, mood problems (feeling depressed/loss of interest), and anxiety. Respiratory symptoms included shortness of breath and cough and cardiovascular symptoms included palpitations.

The questionnaire link was posted on different social media sites (Facebook^®^, Instagram^®^ and WhatsApp^®^) to reach different clusters among the population all around Jordan and from different age groups. Moreover, it was sent *via* email to all students enrolled in Yarmouk University. A paper copy of the questionnaire was also distributed to patients in vaccination centers. To minimize errors in data collection, the respondents with any exclusion criteria characteristics could not proceed to the questions. Finally, after a total number of 23 questions, the respondents were able to submit the answers, and those answers were sent to the drive.

### Data analysis and statistical methods

Descriptive and inferential statistics were used in the statistical analysis. The range, mean, and standard deviation (SD) for the continuous variables, frequencies, cross tabulation, and odds ratio (OR) for categorical variables were calculated. Also, clustered bar charts were used for data visual examination. The chi-squared test of independence was used to investigate the relationship between the two categorical variables. Multiple binary logistic regression was conducted to assess the dependency of different symptoms on gender and age. All presented *p*-values were two-tailed, and *p*-values < 0.05 were considered as statistically significant. All statistical analyses were performed using statistical package SPSS 21.0 (SPSS Inc., Chicago, IL).

Cross tabulations of different symptoms vs. gender and age group (<30 and ≥30 years) are shown in Table 1. The symptoms considered in this study are mood disturbance/depression, fatigue, anxiety, changes in smelling sensation, myalgia, sleep disturbance, palpitation, residual symptoms, weakness, nightmares/flashbacks, shortness of breath, weight loss (>3 Kg), loss of smelling sensation, cough, and loss of taste sensation.

**TABLE 1:** The prevalence of PCS symptoms in the overall population.

Post-COVID-19 symptoms	Prevalence population (*n*, %) *n* = 495	Prevalence of PCS according to gender *n* (%)			Prevalence of PCS according to age *n* (%)		
		Female *n* = 329	Male *n* = 166	*P*-value	Age < 30 years *n* = 207	Age ≥ 30 years *n* = 288	*P*-value
Mood disturbance/depression	294, 59.4%	204 (62%)	90 (54%)	0.059	126 (61%)	168 (58%)	0.579
Fatigue	279, 56.4%	201 (61%)	78 (47%)	**0.003**	123 (59%)	156 (54%)	0.271
Anxiety	234, 47.3%	167 (51%)	67 (40%)	**0.036**	104 (50%)	130 (45%)	0.274
Myalgia	209, 42.2%	153 (47%)	56 (51%)	**0.007**	106 (51%)	103 (36%)	**0.001**
Sleep disturbance	203, 41.1%	141 (43%)	62 (37%)	0.246	83 (40%)	120 (42%)	0.781
Change in smell	202, 40.8%	156 (50%)	46 (30%)	**<0.001**	77 (37%)	125 (47%)	0.524
Palpitation	182, 36.8%	133 (40%)	49 (30%)	**0.018**	76 (38%)	106 (37%)	1.00
Residual symptoms	168, 33.9%	126 (38%)	42 (13%)	**0.002**	72 (35%)	96 (33%)	0.773
Weakness	161, 32.5%	121 (36%)	40 (24%)	**0.004**	78 (38%)	83 (29%)	**0.041**
Nightmares/flashbacks	138, 27.9%	95 (29%)	43 (26%)	0.525	51 (25%)	87 (30%)	0.817
Shortness of breath	138, 27.9%	110 (33%)	28 (17%)	**<0.001**	57 (28%)	81 (28%)	0.919
Weight loss (>3 Kg)	135, 27.3%	94 (29%)	41 (33%)	0.393	63 (30%)	72 (25%)	0.185
Loss of smell sensation	73, 14.7%	55 (17%)	18 (12%)	0.107	33 (16%)	40 (14%)	0.089
Cough	69, 13.9%	49 (15%)	20 (12%)	0.413	28 (14%)	41 (14%)	0.896
Loss of taste sensation	26, 5.3%	21 (6%)	5 (3%)	0.137	11 (5%)	15 (5%)	1.00

Bold values mean significant association with age/gender.

Multiple binary logistic regression has been used to analyze the relationship between predictors and a dichotomous categorical outcome variable. In this paper, multiple binary logistic regression is used to analyze the relationship between each symptom outcome (1: present, 0 absent) and the gender and age variables. The interpretation of Odds Ratio (OR) for gender is that holding the age constant, the odds of symptoms occurring (increased or decreased) by [some percent] for females compared to males.

## Results

### Demographic characteristics

**FIGURE 1:**
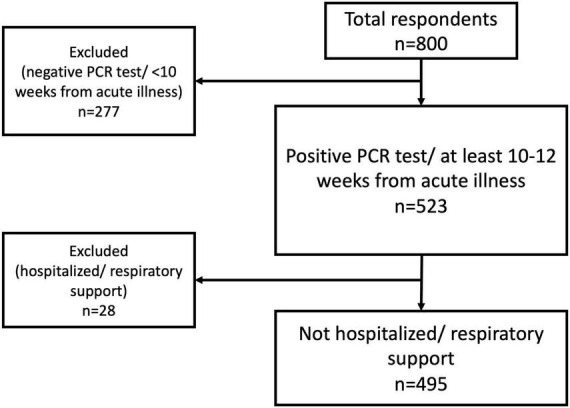
Flow chart of inclusion/exclusion process.

A total of 495 subjects (from 800 overall responders) met the inclusion criteria (Figure 1). The mean (SD) age of the responders was 30.5 ± 10.9 years with a range of 14–70 years. Most of the respondents were females (*n* = 329, 66.4%) (Table 1).

### Prevalence and type of post-COVID-19 syndrome symptoms

In the overall population 83% of patients had at least one PCS symptom and 33.9% had at least one residual symptom persisting after mild or moderate SARS-CoV-2 infection. The prevalence of PCS symptoms in the overall population is reported in Table 1.

In the overall population, 73.3% of patients had at least one psychological consequence of PCS. The most common symptom of PCS was mood disturbance/feeling depressed (59.4%) and fatigue was the second most common symptom (56.4%). Myalgia and weakness were detected in 42.2 and 32.5% of patients, respectively. The weight loss rate occurred in 27.3% of patients. Smelling and taste disorders were relatively rare after COVID-19 (14.7 and 5.3%, respectively). Respiratory symptoms such as breathlessness and cough were detected in 27.9 and 13.9% of patients, respectively. Palpitations were also frequent as PCS symptom in 36.8% of patients.

### Prevalence and type of post-COVID-19 syndrome symptoms by gender and age

The prevalence of PCS symptoms according to gender and age is reported in Table 1. *Gender:* Female reported more PCS symptoms in comparison with male [fatigue (61 vs. 47%, *p* = 0.003), anxiety (51 vs. 40%, *p* = 0.036), palpitation (40 vs. 30%, *p* = 0.018), residual symptoms (38 vs. 13%, *p* = 0.002), weakness (36 vs. 24%, *p* = 0.004), shortness of breath (33% vs. 17, *p* < 0.001), and change in smelling sensation (50 vs. 30%, *p* < 0.001)]. Only myalgia was significantly more frequently reported in male than female (51 vs. 47%, *p* = 0.007). *Age:* No significant differences were found in prevalence of PCS between subjects aged < 30 years/old and ≥ 30 years/old, except for myalgia and weakness which were more frequent in subjects aged < 30 years/old in comparison with those aged ≥ 30 years/old [(51 vs. 36%, *p* = 0.001) and (38 vs. 29%, *p* = 0.041), respectively].

**FIGURE 2:**
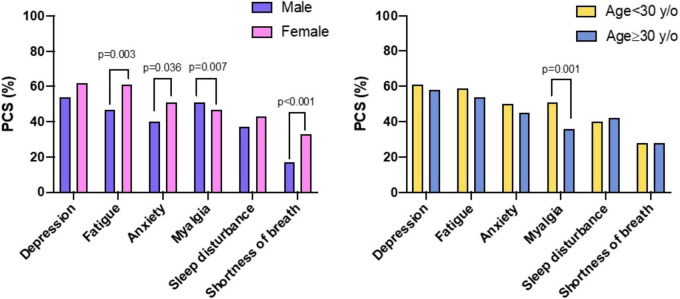
Clustered bar chart of different Post-COVID-19 Syndrome (PCS) symptoms according to gender (left chart) and age (right chart).

Clustered bar chart of different PCS symptoms according to gender and age is shown in Figure 2.

### Risk factors for post-COVID-19 syndrome symptoms

Multiple binary logistic regression analysis has been performed to assess the association between different symptoms with gender and age (Table 2).

**TABLE 2:** Analysis of the association between different symptoms with gender and age.

Outcome	Age			Gender		
	OR	95% CI	*P*-value	OR	95% CI	*P*-value
Mood disturbance/depression	0.998	0.982–1.015	0.818	1.380	0.940–2.003	0.095
Fatigue	1.003	0.986–1.020	0.732	1.768	1.213–2.578	**0.003**
Anxiety	0.992	0.976–1.008	0.319	1.533	1.050–2.238	**0.027**
Change in smell	0.979	0.962–0.997	**0.024**	2.355	1.559–3.560	**<0.001**
Myalgia	1.029	1.012–1.046	**0.001**	1.691	1.143–2.503	**0.009**
Sleep disturbance	0.992	0.975–1.008	0.318	1.272	0.865–1.862	0.218
Palpitation	0.990	0.973–1.007	0.243	1.633	1.088–2.421	**0.016**
Residual symptoms	0.993	0.975–1.010	0.405	1.843	1.209–2.771	**0.004**
Weakness	1.016	0.998–1.033	0.075	1.817	1.192–2.770	**0.005**
Nightmares/flashbacks	0.986	0.967–1.005	0.139	1.172	0.771–1.796	0.462
Shortness of breath	0.995	0.977–1.014	0.621	2.484	1.558–3.961	**<0.001**
Weight loss (>3 Kg)	1.004	0.986–1.022	0.679	1.216	0.787–1859	0.368
Loss of smell sensation	0.996	0.973–1.019	0.714	1.655	0.929–2.899	0.082
Cough	0.992	0.969–1.017	0.533	1.284	0.734–2.239	0.380
Loss of taste sensation	0.998	0.962–1.035	0.906	2.199	0.813–5.938	0.120

CI, confidence interval; OR, Odds Ratio. Bold values mean significant association with age/gender.

*Gender:* Fatigue (*p* = 0.003, OR = 1.768), anxiety (*p* = 0.027, OR = 1.533), palpitation (*p* = 0.016, OR = 1.633), weakness (*p* = 0.005, OR = 1.817), shortness of breath (*p* < 0.001, OR = 2.484), change in smelling sensation (*p* < 0.001, OR = 2.355), myalgia (*p* = 0.009, OR = 1.691) were significantly associated with gender.

*Age:* Myalgia (*p* = 0.001, OR = 1.029) and change in smelling sensation (*P* = 0.024) were significantly associated with age. The loss of baseline physical strength post infection was independently associated (*p* = 0.041) with female gender and age < 30 years.

## Discussion

In the present study, conducted in Jordan among patients after mild to moderate SARS-CoV-2 infection, data indicated that: (1) PCS symptoms were frequent and mainly associated with female gender; (2) psychological symptoms were prevalent; (3) age < 30 years was more likely associated with myalgia, and loss of physical strength.

To our knowledge this is the first study investigating the prevalence of PCS and its different symptoms in patients after mild to moderate SARS-CoV-2 infection in Jordan who were not hospitalized and/or required respiratory support. This was a prospective study enrolling a significant number of patients considering the overall population in the country. Further, we were able to identify risk factors for PCS in a specific geographical area with local style of life and support of patients. Currently available data reporting PCS prevalence after SARS-CoV-2 infection are related to severe infection requiring hospitalization and/or need of mechanical ventilation ranging from 85 to 87.4% (8, 9). In the present study we found that the overall prevalence of PCS symptoms is 83% which is not consistent with those previously reported in other geographical areas such as Europe (44%) and North America (31%) (10). Among different PCS symptoms, psychological (mood disturbance/feeling depressed) and fatigue symptoms were the most frequently reported in the present study in parallel with previous reports (11); this may be attributed to the fact that patients have been in quarantine for 14 days and/or feared possible worsening of infection during the time of acute illness. Premraj et al. (12) reported fatigue as the most frequent symptom of PCS followed by brain fog, sleep disturbances and memory issues. Myalgia and palpitations were also frequent in the present study which was not in agreement with previous studies [5.9% for myalgia (13) and 8.3% for palpitations (14)]. Finally, respiratory symptoms, including cough (14%) and breathlessness (28%) were less frequent than expected and in comparison, with other studies (15, 16) where respiratory symptoms are more frequent (30–50% for breathlessness).

We found that PCS symptoms, including myalgia and weakness, were highly prevalent in female gender and age < 30 years. This information may help to optimize healthcare monitoring and support after mild to moderate COVID-19 infection. On the contrary, Oronsky et al. (17), found that older age was a risk factor for PCS symptoms. Our findings were the opposite from those reported by Peghin et al. (18), who found no association between age and PCS symptoms after COVID-19. A previous study performed in the Middle East has pointed to female gender as a risk factor for PCS (19), suggesting a possible relation with Arabic culture, as females are used to take care of family members when they are infected. On the other hand, a relatively significant increase in PCS symptoms among females in UK suggests a possible biological relationship (20). Daily behaviors and environment are hypothesized to affect the probability of developing PCS symptoms. A previous observational study noted that isolation, financial status, exercise, temperature, and humidity may increase the risk of PCS symptoms (21). The presence of comorbidities is also a well-established risk factor for PCS, as multiple studies indicated that the presence of pre-existing medical conditions (*P* = 0.003) increases the potential of having PCS. In addition, having hypertension (odds ratio (OR) = 1.3, *P* = 0.018), obesity (OR = 2.31, *P* = 0.002), a psychiatric condition (OR = 2.32, *P* = 0.007), or an immunosuppressive condition (OR = 2.33, *P* = 0.047) corresponded with the greatest odds of not returning to “usual health.” (22). Having blood group O was associated with an increased risk of developing PCS, as group O showed a sixfold increased risk of PCS, compared to non-O (23). Smoking, low economic status, and full vaccination prior COVID-19 were also considered risk factors in patients enrolled in a single-center longitudinal study (24). Sudre et al. (25) demonstrated that 13.3% of participants reported symptoms lasting ≥ 28 days, 4.5% ≥ 8 weeks, and 2.3% ≥ 12 weeks. Therefore, physical, and mental rehabilitation of PCS play a relevant role to facilitate the healing process (26, 27).

In the open-ended question, people reported having the following symptoms: headache, memory problems, hair loss, joint pain and lower back pain (*n* = 11, 8, 6, 5, and 4, respectively), and these symptoms were major symptoms of PCS in multiple studies (28–30). Some rare manifestations of the post-viral syndrome have also been reported; according to Goërtz et al. (31) symptoms like eye problems, ear pain, red spots on toes/feet, and vomiting were noticed.

### Limitations

This study has several limitations that should be addressed. First, to facilitate the survey and response rate several issues related to PCS have not been specifically addressed. Second, this survey collected data during a single time point, limiting the validity of temporal association. Third, a control of response accuracy was not feasible. Fourth, comorbid conditions were not investigated which might affect the prevalence of post-COVID symptoms. Fifth, a potential bias due to the survey strategy was encountered as it is more likely that symptomatic individuals were more prone to answer the survey than asymptomatic ones, explaining the high (83%) positivity compared to the literature available.

## Conclusion

PCS is highly prevalent in COVID-19 survivors in Jordan, especially in females and patients with comorbidities. Planning physical and mental rehabilitation services is recommended for those patients with PCS symptoms after mild to moderate COVID-19.

## Data availability statement

The raw data supporting the conclusions of this article will be made available by the authors, without undue reservation.

## Ethics statement

The studies involving human participants were reviewed and approved by the International Review Board (IRB) of Yarmouk University number: IRB/2022/9. Written informed consent for participation was not required for this study in accordance with the national legislation and the institutional requirements.

## Author contributions

LA-H contributed to the writing—review and editing, supervision, and project administration. MN conceived the research questions, performed statistical analysis, designed the questionnaire, and data input. HA-G performed statistical analysis and data input. AA contributed to the review, editing, and style. MS performed statistical analysis. DB and PP contributed to the writing—review and editing and supervision. All authors contributed to the article and approved the submitted version.
