# Supplementary material for: Post-COVID-19 syndrome symptoms after mild and moderate SARS-CoV-2 infection
Source: Front Med (Lausanne). 2022 Oct 3;9:1017257. doi: 10.3389/fmed.2022.1017257 (PMC9573938; doi:10.3389/fmed.2022.1017257)
Supplement: Supplementary file 1 [file Table_1.DOCX]

**Supplementary material**

**Newcastle post-COVID syndrome Follow Up Screening Questionnaire**

**(available from:** [**https://postcovidsyndromebsol.nhs.uk/images/Content/Newcastle_post_Covid_Screening_Tool.pdf**](https://postcovidsyndromebsol.nhs.uk/images/Content/Newcastle_post_Covid_Screening_Tool.pdf)**)**

Name....................................................
Date of Positive Swab.........................
Date of Onset of symptoms............................
Date of Discharge (for hospital admissions) ................................

Date of call _____________
Person phoning ________________

Role _______________

Level of respiratory support during acute Illness:

- ICU
- Intubated ICU
- Not intubated
- Enhanced Respiratory support (e.g. CPAP)
- Supplemental oxygen
- Managed in the community

**Have you made a full recovery or are you still troubled by symptoms?**

• Symptoms
• Full Recovery

**Are you more breathless now than you were before your COVID illness?**

**Is this more than you would have expected by now? OR**

**Do you think you are on your way back to full fitness?**

**Do you feel fatigued (worn out/lacking energy or zest) compared with how you were before your COVID illness?**

**Is this more than you would have expected by now? OR**

**Do you think you are well on your way back to full fitness?**

**Do you have a cough (different from any cough you may have had before COVID19)?**

**• Yes • No**

**Do you get any palpitations? (sense that you can feel your heart pounding or racing)**

**• Yes • No**

**How is your physical strength? Do you feel so weak that it still limiting what you can do (more than you were pre your COVID illness)?**

**• Yes • No**

**Do you have any myalgia (‘aching in your muscles’)?**

**• Yes • No**

**Do you have anosmia (‘no sense of smell’)?**

**• Yes • No**

**Have you lost your sense of taste?**

**• Yes • No**

**Is your sleep disturbed (more than it was pre-COVID)?**

• Yes • No

**Have you had any nightmares or flashbacks?**

• Yes • No

**On your mood**

Is your mood low/do you feel down in the dumps/lacking in motivation/no pleasure in anything?

• Yes • No

**Do you find yourself feeling anxious/worrying more than you used to?**

• Yes • No

**Have you lost weight (> 1⁄2 stone, 3 Kg) since your COVID illness?**

• Yes • No

**Any other symptoms**

**___________**
